# Variations in phyllosphere microbial community along with the development of angular leaf-spot of cucumber

**DOI:** 10.1186/s13568-019-0800-y

**Published:** 2019-05-27

**Authors:** Luyun Luo, Zhuo Zhang, Pei Wang, Yongqin Han, Decai Jin, Pin Su, Xinqiu Tan, Deyong Zhang, Hamid Muhammad-Rizwan, Xiangyang Lu, Yong Liu

**Affiliations:** 1 College of Bioscience & Biotechnology, Hunan Agricultural University, Changsha, China; 2 Key Laboratory of Pest Management of Horticultural Crops of Hunan Province, Hunan Plant Protection Institute, Hunan Academy of Agricultural Science, Changsha, China; 3 Chinese Academy of Sciences Key Laboratory of Environmental Biotechnology, Research Center for Eco-Environmental Sciences, Chinese Academy of Sciences, Beijing, China

**Keywords:** Phyllosphere, Microbial community, Lesion coverage rate, α Diversity, Plant-specific microbe

## Abstract

The phyllosphere is colonized by a wide variety of microorganisms including epiphytes, plant-pathogenic fungus, bacteria, as well as human or animal pathogens. However, little is known about how microbial community composition changes with the development of angular leaf-spot of cucumber. Here, 18 mixed samples were collected based on the lesion coverage rate (LCR) of angular leaf-spot of cucumber from three disease severity groups (DM1: symptomatic-mild, DM2: symptomatic-moderate, DM3: symptomatic-severe). In our study, the microbial community structure and diversity were examined by Illumina MiSeq sequencing. A significant differences was observed in α diversity and community structure among three disease severity groups. The phyllosphere microbiota was observed to be dominated by bacterial populations from Proteobacteria, Actinobacteria, and Firmicutes, as well as fungal species from Ascomycota and Basidiomycota. In addition, some plant-specific microbe such as *Sphingomonas*, *Methylobacterium*, *Pseudomonas*, and *Alternaria* showed significant changes in their relative abundance of population. The LCR was correlated negatively with *Sphingomonas*, *Methylobacterium*, *Quadrisphaera*, and *Lactobacillus*, whereas correlated positively with *Pseudomonas* and *Kineococcus* (p < 0.05). The LCR was negatively correlated with *Alternaria* and *Arthrinium* of the fungal communities (p < 0.05). Molecular ecological networks of the microbial communities were constructed to show the interactions among the OTUs. Our current results indicated that the competitive relationships among species were broken with the development of angular leaf-spot of cucumber. The microbial community composition changed over the development of angular leaf-spot of cucumber. The result of molecular ecological networks indicated that the overall bacterial community tends toward mutualism from the competition. The development of angular leaf-spot of cucumber affected the ecosystem functioning by disrupting the stability of the microbial community network. This work will help us to understand the host plant-specific microbial community structures and shows how these communities change throughout the development of angular leaf-spot of cucumber.

**Electronic supplementary material:**

The online version of this article (10.1186/s13568-019-0800-y) contains supplementary material, which is available to authorized users.

## Introduction

Vegetables not only act as energy regulators for human, but also a major part of the human diet with great nutritive values. More than a decade ago, cucumbers were planted in an area of around 156,300 ha around the world with a total production of 26,582 tons (Ram 2002). Cucumber yield and quality are affected by many fungal, bacterial and viral diseases. The angular leaf spot caused by *Pseudomonas syringae* pv. lachrymans (Young et al. 1978), is distributed worldwide and caused heavy economic losses under favorable climatic conditions by decreasing the yield as well as the quality of the cucumber (Pohronezny et al. 1977).

The phyllosphere is colonized by specific microbial communities as a vital plant-associated habitat (Vorholt 2012; Bringel and Couée 2015). Leaves of plant are colonized by potential microbial communities including bacteria, fungi, protists, and viruses. There are numbers of bacteria colonizing in leaf surface ranging from 10^6^ to 10^7^ per cm^2^ (Vorholt 2012). The phyllosphere may form complex microbial consortia that can be beneficial, pathogenic, or antagonistic for the host plant, and contribute greatly to plant health and yield through complex plant-microbial interactions (Vorholt 2012; Bulgarelli et al. 2013; Brader et al. 2017). The phyllosphere microbial community structure were affected strongly due to changes in the relative abundance of those “key” microbes induced by abiotic or biotic factors. For example, it can be influenced by the plant species, season, geographical location, and different environmental conditions (Whipps et al. 2008; Knief et al. 2010; Wellner et al. 2011; Rastogi et al. 2012; Copeland et al. 2015; Ding and Melchner 2016).

Phyllosphere microbes can affect host fitness through the production of plant hormones and protection from pathogen colonization (Innerebner et al. 2011; Ritpitakphong et al. 2016). In order to adapt to host plants, phyllosphere microorganisms can affect community diversity and plant population (Clay and Holah 1999; Bradley et al. 2008), as well as ecosystem functions (Rodriguez et al. 2009; McGuire and Treseder 2010; Allison and Treseder 2011). The pathogens are expected to compete with native plant-associated microbes when they reach plant surfaces (Brandl et al. 2013). At the same time in the phyllosphere, these microbes will also face harsh environmental conditions including nutrient limitations, UV radiation, as well as lack of free water availability (Colla et al. 2017). All host plants are capable of activating an effective generic defense response against a wide range of microbes (Pieterse et al. 2014). Consequently, microbes usually have specialized structure to help them move towards the plant surfaces. Although the microbial community structure in the leaves has been elucidated, there is still a lack of knowledge about microbial interactions among microbial composition (Hacquard and Schadt 2015). In particularly, it remains unclear that how the establishment of microbial community were affected by competition and mutualism among microbes. It is likely that members of the microbial communities associated with plant have evolved complex strategies for interacting in complex microbial communities to maintain specific host niches.

At present, the research is mainly focused on the culturable part of leaf microflora but it is limited on the understanding of the functional traits of phyllosphere microbes. With the development of next-generation sequencing technology and related computational analysis tools, we can now perform the further investigations (Bulgarelli et al. 2012; Lundberg et al. 2012). The previous studies indicated that the bacterial communities of different plant species are plant-host dependent (Knief et al. 2010; Redford et al. 2010; Vorholt 2012). These findings suggested that the plant host and colonizers might have a selection for each other. However, the underlying processes of microbial population dynamics remain to be elucidated. In this study, our aim was to (1) compare the inter-individual and inter-specific variation of phyllosphere microbial communities; (2) characterize the composition of phyllosphere microbial communities at different disease severities of angular leaf-spot of cucumber; and (3) determine the correlation between disease severity of angular leaf-spot of cucumber and phyllosphere population.

## Materials and methods

### Experimental design

The experiment was performed in the base of LangLi town, Changsha, Hunan Province, China (28°16′92″N, 113°14′29″E) during June 2016. The cucumber (Suyan 10) was planted in six adjacent greenhouses, damaged by angular leaf-spot of cucumber for a long time. Leaf samples were collected from cucumber (Suyan 10) plants based on the proportion of lesion area of angular leaf-spot of cucumber (0 < DM1 < 30%, 30% < DM2 < 50% and DM3 > 50%). The applied classification method of disease severity was according to the National standard in China (GB/T 17980.30-2000) with slight modifications. According to three different disease severities, 10 cucumber leaves of same disease severity with the same size at the fruiting stage were collected by five-point sampling method in each greenhouse, respectively. Then the leaf samples of same disease severity from the same greenhouse were mixed and transferred to the laboratory at refrigerated temperature. For each leaf, sampling was done by using five-point sampling within an area of 40 m^2^. Every sterile bag contained 10 leaves which were cut into tiny pieces and mixed before subsequent processing. The phyllosphere microbes were collected as described by Xie et al. reported (Xie et al. 2015; Delmotte et al. 2009; Redford and Fierer 2009), with slight modifications. In brief, 10 g of leaves were submerged in a 250 mL sterile conical flask with 100 mL of PBS containing 0.01% Tween-80. The flask was then shaken at 250 rpm for 30 min at 28 °C, and subjected to ultrasonic-solid for 10 min. The microbes were filtered by a 0.22 μm filter microfiltration membrane using the air pump filtration, and were stored at − 80 °C for subsequent DNA extraction.

### DNA extraction and purification

DNA was extracted from phyllosphere samples according to the manufacturer’s protocol using the MP FastDNA^®^ SPIN Kit for soil (MP Biochemicals, Solon, OH, USA). PCR amplicon libraries concentration were diluted to 30 ng/μL for each sample. The V5–V6 region of the bacterial 16S rRNA gene was amplified by using the specific primer 799F (AACMGGATTAGATACCCKG) and 1115R(AGGGTTGCGCTCGTTG). The eukaryotic primers gITS7 (5′-GTGARTCATCGARTCTTTG-3′) (Ihrmark et al. 2012) and ITS4 (5′-TCCTCCGCTTATTGATATGC-3′) (White et al. 1990) with a unique 6 nt barcode were included as the modification in the forward and reverse primer, respectively. The bacterial and fungal ITS regions were amplified as previously described by Kong et al. (2018, 2019). PCR products were purified with an E.Z.N.A.^®^ Gel Extraction Kit, pooled in equimolar amounts using Qubit (CA, USA). And mixed PCR products were sequenced (2 × 250 bp) on an Illumina MiSeq platform by ANNOROAD Gene Technology Co., Ltd. (Beijing, China) according to standard protocols.

### Processing of sequencing data

Raw sequence data reads were processed with an in-house pipeline (http://mem.rcees.ac.cn:8080) which includes a series of bioinformatics tools. In brief, a separate sample was generated according to different 12-bp barcodes and primers, allowing for one mismatch. Paired-end reads with at least 30 bp overlap were combined by the FLASH program (Magoc and Salzberg 2011). The combined sequences (quality score < 20) were filtered by Btrim program (Kong 2011). Then the sequences with either an ambiguous base or < 200 bp were discarded. The UPARSE algorithms were used to detect and remove chimera sequences (Edgar 2013). Low abundance OTUs (≤ 1 count) were eliminated from the OTU table which was clustered and generated at a 97% similarity from all sequences. The microbial representative sequences for each OTU were assigned to taxonomic groups using the RDP Classifier database (Silva database 132 version) and UNITE database (Version 12.01.2017) (Abarenkov et al. 2010), respectively. The data were resampled randomly with the lowest sequence number (10,217 for bacteria and 13,342 sequences for fungi). The resampled OTU table was used for the subsequent analysis. In this study, all the microbial raw sequences were deposited in the SRA database short-read archive PRJNA503587.

### Network construction and analysis

Phylogenetic molecular ecological networks (pMENs) of the three groups (DM1, DM2 and DM3) were constructed based on the Spearman rank correlation matrix using by molecular ecological network analysis pipeline (MENA, http://ieg4.rccc.ou.edu/mena/login.cgi) (Deng et al. 2012; Zhou et al. 2010a, b, 2011). The process was described as Deng et al. (2012) reported. Firstly, only the OTUs appeared in more than half samples for each group were kept without log-transferring prior to obtaining the Spearman rank correlation matrix with a series of thresholds from 0.01 to 0.95 with 0.01 interval. Then only the correlations above a specific threshold (0.86 for bacteria and 0.92 for fungi) were kept for calculating the network eigenvalues. The network plots were visualized with the software Cytoscape 3.6.0.

### Statistical analysis

The difference between α diversity indices (Shannon index, inverse Simpson index, richness (observed_richness), Chao’s estimated richness (Chao1) and relative abundance of the taxonomic subgroups) was assessed by performing a one-way ANOVA followed by Duncan’s multiple range test (p < 0.05). Correlation analysis between lesion coverage rate (LCR) and α diversities along with some special genera analysis was also analyzed with Pearson, Kendall, and Spearman method. The above statistical analyses were performed using the software IBM SPSS (Version 21.0) for Windows.

Detrended correspondence analysis (DCA) were performed in subsequent analysis to compare the microbial community composition difference between two groups. Venn diagram analysis showed the shared and united OTU among three groups. The microbial community composition difference between two groups were evaluated by using Nonparametric multi-response permutation procedures (MRPP), analysis of similarities (ANOSIM) and Non-parametric permutational multivariate analysis of variance of the Adonis function (ADONIS) (Anderson 2001; Dixon 2003). The above analysis was performed using vegan package in R package (v.3.2.5). Analysis of fungal trophic modes annotation was performed with FunGuild (Nguyen et al. 2016).

## Results

### The α diversity and community structure of microorganism under different disease severities

**Fig. 1:**
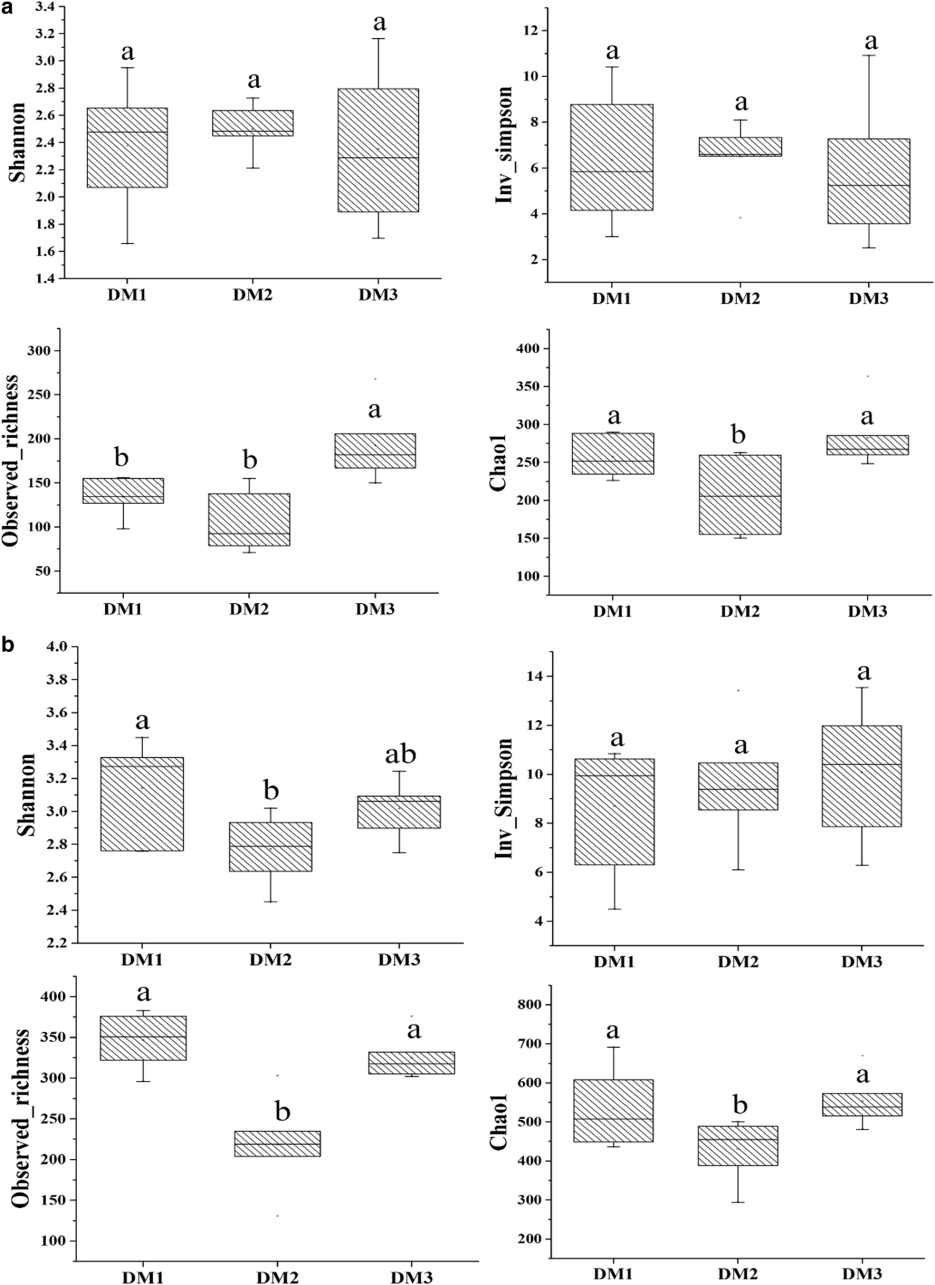
Summary of α diversity indices under different disease severities. The α diversity indices including shannon, Inv_simpson indice, Observed_richness and Chao1 in bacteria (**a**) and fungi (**b**). The data were analyzed based on a one-way ANOVA followed by Duncan’s multiple range test at p < 0.05. DM1, DM2 and DM3 represent the three disease severities of angular leaf-spot of cucumber, respectively. *DM1* symptomatic-mild, *DM2* symptomatic-moderate, *DM3* symptomatic-severe

In this study, 18 mixed phyllosphere samples were collected and sequenced. A total of 883,665 bacterial and 657,814 ITS raw sequences were obtained from the high-throughput sequencing. After completed data analysis, these high-quality sequences were classified into 404 bacterial and 948 ITS operational taxonomy units (OTUs) at a 97% similarity level, respectively. The rarefaction curves indicated that the number of sequences for all samples reached the sequencing depths (Additional file 2: Figure S1). The α diversity indices including observed_richness, Chao1, Shannon index and Inverse Simpson index were shown in Fig. 1. For bacterial α diversity, Shannon and Inv_simpson index first increased then decreased from DM1 to DM3, while richness and Chao1 index first decreased then increased significantly. For fungal α diversity, all diversity indices increased from DM1 to DM2 and then decreased from DM2 to DM3; while the DM2 group showing lowest fungal diversity.

**Fig. 2:**
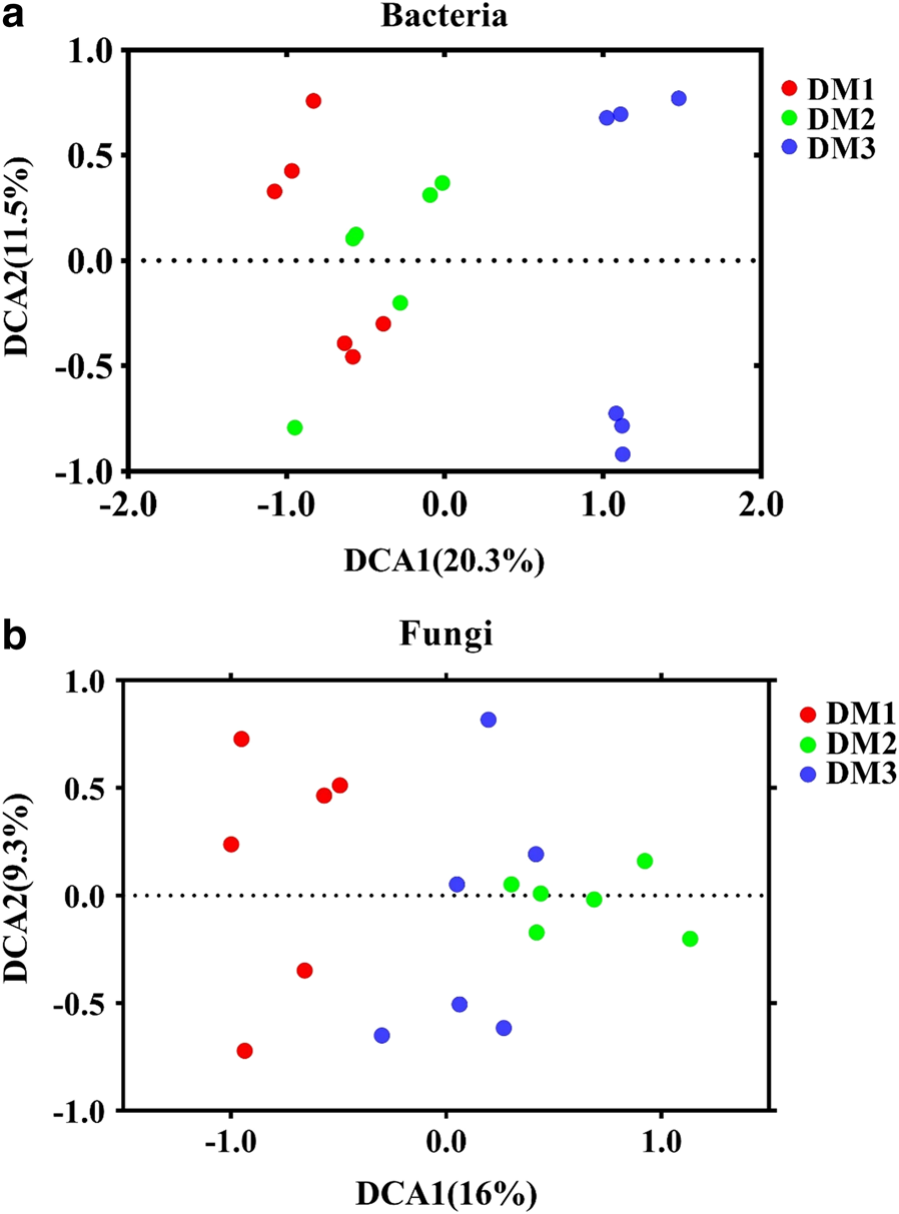
Detrended correspondence analysis (DCA) under different disease severities. DM1, DM2 and DM3 represent the three disease severities of angular leaf-spot of cucumber, respectively. *DM1* symptomatic-mild, *DM2* symptomatic-moderate, *DM3* symptomatic-severe

The detrended correspondence analysis (DCA) was used to measure the dissimilarity of microorganism communities among all groups (Fig. 2). In general, the microorganism community within the phyllosphere samples were clearly separated under different disease severity. In addition, the dissimilarity test also showed that differences in the phyllosphere communities among the three groups were significantly different (p < 0.05) (Additional file 1: Table S1).

### The population analysis under different disease severities

The shared and unique OTU numbers are shown in the Venn diagram (Additional file 2: Figure S2). These shared OTUs accounted for more than half the total OTUs in bacteria (232, 57.43%) and fungi (514, 54.22%). The DM3 and DM1 group had the highest number of unique OTUs in bacterial (14.36%) and fungal (8.54%) microbial communities, respectively.

In totally, 13 bacterial phyla and 4 fungal phyla were detected (Additional file 2: Figure S3). Proteobacteria, Actinobacteria, and Firmicutes accounted for more than 98% of all high-quality bacterial sequences in DM1 (98.98%), DM2 (99.34%) and DM3 (99.17%). While in case of fungal phyla, members from Ascomycota and Basidiomycota accounted for 89.67% (DM1), 97.06% (DM2) and 87.33% (DM3), respectively (Additional file 2: Figure S3). At the class level, a total of 25 bacterial and 24 fungal classes were detected (Additional file 2: Figure S4).

Microbial genera with relative abundance greater than 1% are shown in Additional file 1: Table S2 and main genera are shown in Additional file 2: Figure S5. At the bacterial genus level, it was observed that the relative abundance of *Quadrisphaera* decreased significantly from DM1 to DM2 and then kept stable. Relative abundance of *Sphingomonas* and *Microbacterium* increased from DM1 to DM2 and then decreased from DM2 to DM3. Relative abundance of *Pseudomonas* increased while *Methylobacterium* and *Curtobacterium* decreased from DM1 to DM3. Relative abundance of *Kineococcus* kept stable from DM1 to DM2 and then increased significantly from DM2 to DM3. At the fungal genus level, it was observed that the relative abundance of *Phoma* and *Davidiella* increased slightly from DM1 to DM3 while the relative abundance of *Alternaria* decreased. Relative abundance of *Sporobolomyces*, *Pseudozyma*, and *Aureobasidium* increased from DM1 to DM2 and then decreased from DM2 to DM3.

### Correlation analysis between lesion coverage rates (LCR), α diversities index and dominant genera

In case of the bacterial community, the LCR had a significant positive correlation with Observed_richness (p < 0.05, Table 1) while had no correlation with Shannon index, Inv_Simpson index and Chao1 (p > 0.05, Table 1). There was no correlation between LCR and fungal diversity indices under different disease severities (p > 0.05, Table 2). The LCR had a significant negative correlation with *Sphingomonas*, *Methylobacterium*, *Quadrisphaera*, *Lactobacillus*, whereas significant positive correlation with *Pseudomonas* and *Kineococcus* of the bacterial population (p < 0.05, Table 1). Meanwhile, the LCR was negatively correlated with fungal populations of *Alternaria* and *Arthrinium* (p < 0.05, Table 1).

**Table 1:** Correlation analysis between lesion coverage rate (LCR) and α diversities index and main genera of bacterial communities

Factors	Pearson	Spearman	Kendall
α Diversity			
Shannon	0.0259	0.144	0.0528
Inv_Simpson	− 0.0354	0.03	0.0264
Observed_richness	0.62**	0.592**	0.447*
Chao1	0.337	0.27	0.211
Main bacteria genera			
*Sphingomonas*	− 0.513*	− 0.562*	− 0.396*
*Microbacterium*	0.103	0.156	0.0924
*Methylobacterium*	− 0.693**	− 0.741**	− 0.568**
*Curtobacterium*	− 0.461	− 0.361	− 0.277
*Pseudomonas*	0.573*	0.508*	0.383*
*Kineococcus*	0.623**	0.673**	0.436*
*Aureimonas*	− 0.289	− 0.248	− 0.146
*Quadrisphaera*	− 0.483*	− 0.535*	− 0.391*
*Novosphingobium*	0.761**	0.581*	0.417*
*Hymenobacter*	− 0.269	− 0.225	− 0.154
*Bacillus*	0.339	0.131	0.0867
*Lactobacillus*	− 0.616**	− 0.718**	− 0.539**

* p < 0.05, ** p < 0.01

**Table 2:** Correlation analysis between lesion coverage rate (LCR) and α diversities index and main genus of fungal communities

Factors	Pearson	Spearman	Kendall
α Diversity			
Shannon	− 0.161	− 0.334	− 0.224
Inv_Simpson	0.186	− 0.0445	− 0.0396
Observed_richness	− 0.0474	− 0.202	− 0.126
Chao1	0.191	0.183	0.172
Main fungal genera			
*Sporobolomyces*	0.23	0.377	0.198
*Davidiella*	0.396	0.522	0.37
*Phoma*	0.363	0.299	0.211
*Alternaria*	− 0.564**	− 0.624**	− 0.444*
*Pseudozyma*	0.238	0.402	0.251
*Aureobasidium*	− 0.384	− 0.457	− 0.298
*Ascomycota_unidentified_1_1*	0.136	− 0.133	− 0.066
*Periconia*	0.368	0.312	0.179
*Exobasidiomycetes_unidentified_1*	− 0.229	− 0.0372	− 0.0331
*Pleosporales_unidentified_1*	− 0.327	− 0.432	− 0.311
*Tremellomycetes_unidentified_1*	− 0.228	− 0.406	− 0.298
*Chaetothyriales_unidentified_1*	− 0.382	− 0.363	− 0.192
*Arthrinium*	− 0.624**	− 0.613**	− 0.444*

* p < 0.05, ** p < 0.01

### Molecular ecological networks under different disease severities

**Fig. 3:**
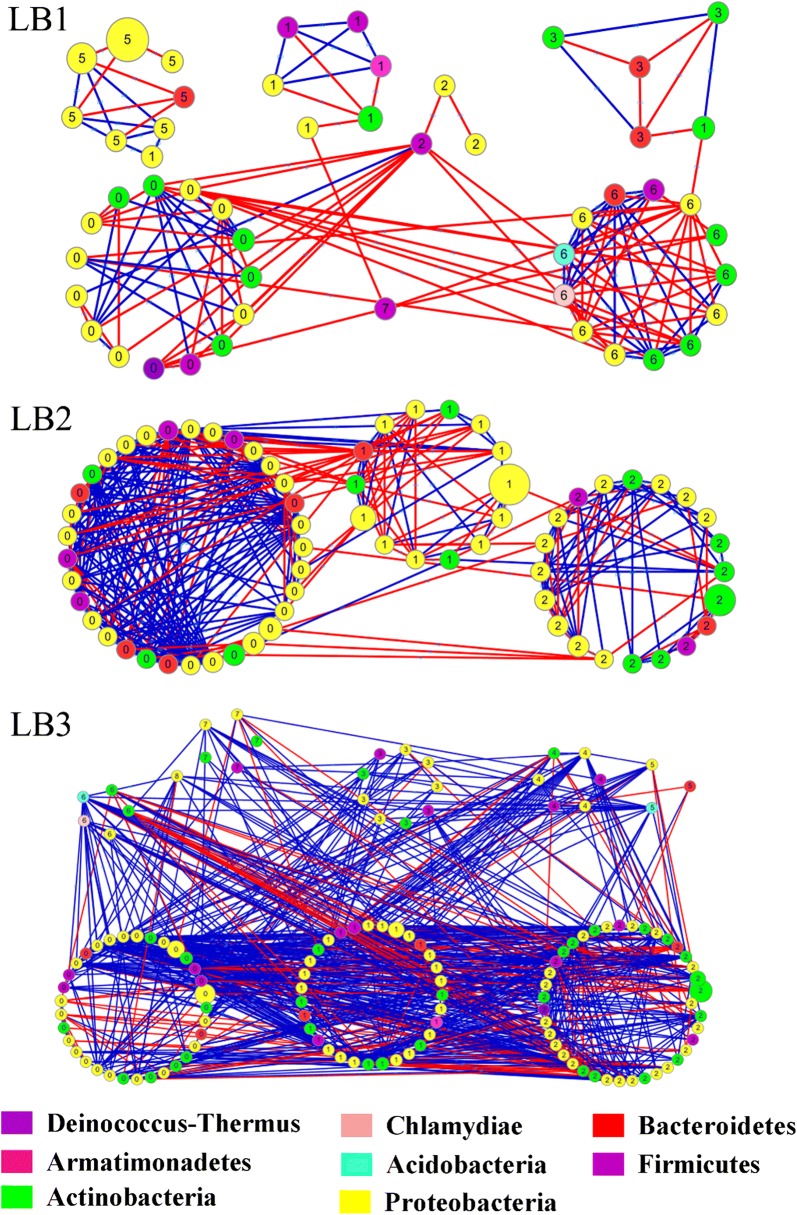
Phylogenetic molecular ecological networks (pMENs) of bacterial communities under different disease severities. Modules with > 5 nodes were obtained for bacterial groups (LB1, LB2 and LB3), respectively. The links between two nodes show the correlation (red: positive, blue: negative). The size of circle indicate the relative abundance of the OTU. The number in the center of circle represents the modules to which these OTUs belongs

**Fig. 4:**
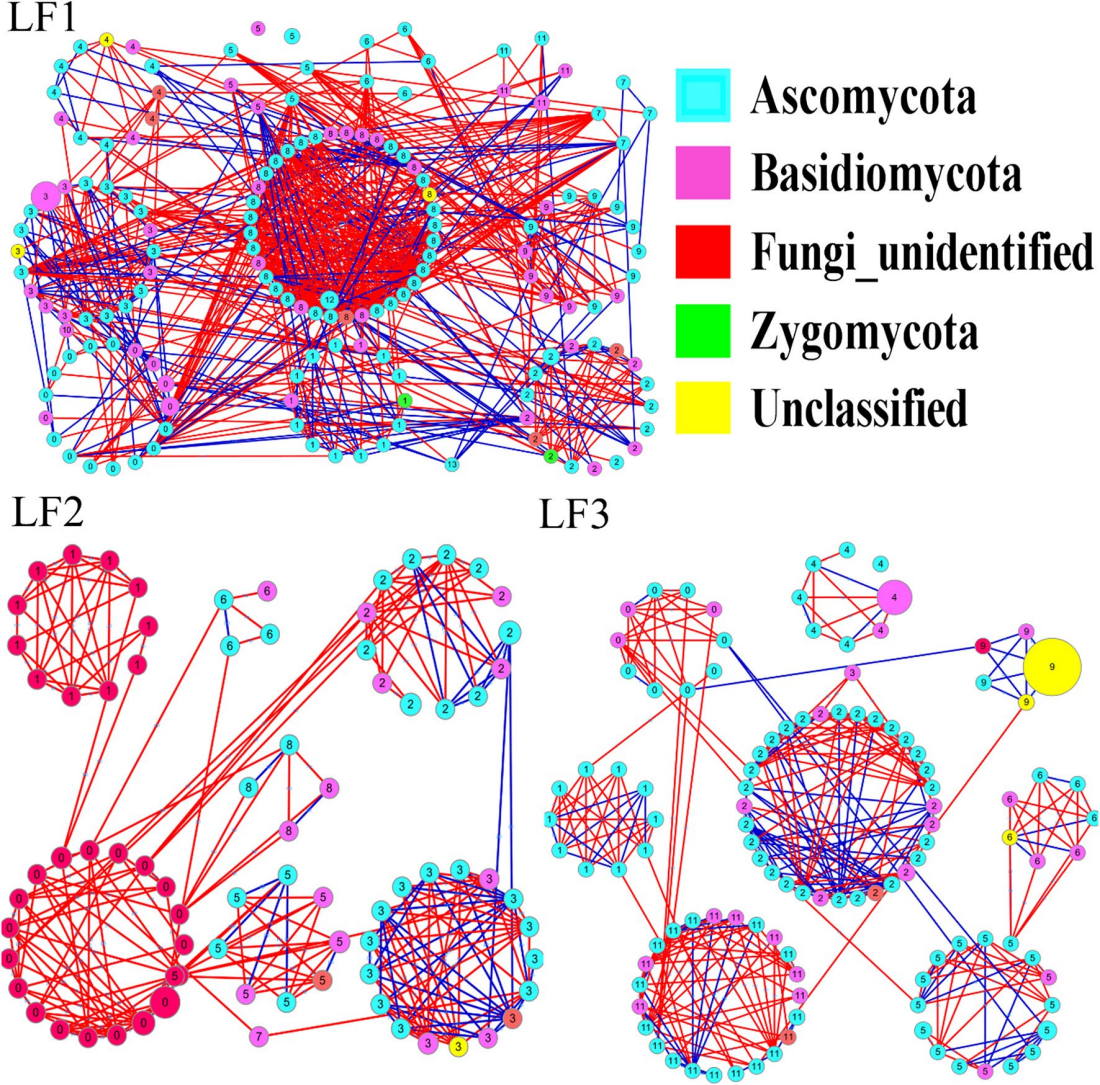
Phylogenetic molecular ecological networks (pMENs) of fungal communities under different disease severities. Modules with > 5 nodes were obtained for fungal groups (LF1, LF2 and LF3), respectively. The links between two nodes show the correlation (red: positive, blue: negative). The size of circle indicate the relative abundance of the OTU. The number in the center of circle represents the modules to which these OTUs belongs

The phylogenetic molecular ecological networks (pMENs) of the bacterial and fungal communities constructed in our study to show the interactions among the OTUs are summarized in Table 3. The result suggested that many of the microbes had a few connections while only few had many connections with others. For bacterial communities, the group DM3 had the highest average connectivity (avgK:9.239) while the group DM1 had the lowest average connectivity (avgK:4.382). For fungal communities, the highest average connectivity was observed in the group DM1 (avgK:5.941) and lowest average connectivity in the group DM3 (avgK:4.432). The overall pMENs of the three groups can be visualized in Figs. 3 and 4, respectively. Modules were identified using fast greedy modularity optimization. A total 3, 8, 6 and 11, 7, 8 modules with > 5 nodes were obtained for DM1, DM2, and DM3 group in bacterial and fungal compositions, respectively. The nodes and links for all three groups i.e. DM1 (306, 909), DM2 (180, 432), and DM3 (296, 656) were obtained in this study.

**Table 3:** The properties of the empirical and random networks under different disease serverity

Taxonomy	Groups	Empirical network						Random networks (100)		
		RMT threshold	nodes	links	Average degree (avgK)	Average clustering coefficient (avgCC)	Modularity (fast_greedy)	Average clustering coefficient (avgCC)	Average path distance (GD)	Modularity (fast_greedy)
Bacteria	DM1	0.86	131	287	4.382	0.245	0.532	0.045 ± 0.012	3.395 ± 0.051	0.439 ± 0.011
	DM2	0.86	95	388	8.168	0.393	0.674	0.146 ± 0.015	2.492 ± 0.034	0.257 ± 0.009
	DM3	0.86	197	910	9.239	0.4	0.482	0.090 ± 0.008	2.678 ± 0.020	0.264 ± 0.007
Fungal	DM1	0.92	306	909	5.941	0.255	0.742	0.036 ± 0.006	3.367 ± 0.029	0.372 ± 0.006
	DM2	0.92	180	432	4.8	0.257	0.768	0.033 ± 0.008	3.435 ± 0.037	0.424 ± 0.009
	DM3	0.92	296	656	4.432	0.193	0.79	0.018 ± 0.006	3.907 ± 0.035	0.464 ± 0.007

DM1, DM2 and DM3 represent the three disease severities of angular leaf-spot of cucumber, respectively

*DM1* symptomatic-mild, *DM2* symptomatic-moderate, *DM3* symptomatic-severe

For bacterial communities, the number of the nodes in three networks belonged to phylum Actinobacteria and Proteobacteria. And most of the nodes were from Ascomycota, Basidiomycota, and some unidentified species of the fungal population. Most of the interactions between the OTUs were positive (42.16–74.4%), and the number of positive interactions increased from DM1 to DM3 in bacteria composition. At same time, a number of negative interactions (55.1–63.26%) were observed among fungal populations.

The topological roles of the OTUs identified in three networks are visualized in Fig. S6. The numbers of the OTUs (97.84%) were peripherals whose links mainly stayed within their respective modules. The OTUs of bacteria (2.09%) and fungi (2.22%) were observed to be generalists. From bacterial communities, 0.17% of OTUs were module hubs (nodes that are connected with nodes within their modules, Zi > 2.5) and 1.92% of OTUs that were connectors (nodes that connected with several modules, Pi > 0.62). In the fungal communities, 0.42% and 1.79% of OTUs were module hubs and connectors, respectively. In addition, all the module hubs were from DM2 and DM3 while all connectors were from all three groups. These OTUs associated with module hubs and connectors are shown in Additional file 1: Table S3.

## Discussion

The phyllosphere is colonized by a wide variety of microorganisms including plant pathogenic microbes and other kinds of pathogens (Lindow and Brandl 2003). The colonization of phyllosphere by microbes is controlled by many factors such as factors from plants, microbes and natural environment. And these microbes can deposit on the surfaces of the leaf by means of many pathways. The microbes colonizing the leaves serving as pathogens or growth promoters may have the neutral, negative, or positive impact on their host plants (Kinkel 1997; Whipps et al. 2008). Therefore, it is crucial to study the effect of pathogens from the phyllosphere microbial communities, especially from the dominant microbiome. It is suggested that the abundance of dominant members were associated with plant-associated microbial communities and it is an important factor to determine the healthy community state (Shade and Handelsman 2012). Utilizing the beneficial potential of the plant microbiome to mitigate the hazards of major crop diseases is becoming a sustainable way to improve agricultural production (Berg et al. 2013; Gopal et al. 2013; Bakker et al. 2012). In our study, we conducted a field experiment to study the dynamic change of phyllosphere microbial communities by an Illumina MiSeq-based approach, under the different stage of angular leaf-spot of cucumber.

The phyllosphere microbiome is crucial for leaf biological processes and ecosystem functions (Ortega et al. 2016). The microbial communities composition and structure were observed to be strongly affected by the plant pathogenic microbe (Rastogi et al. 2012; Bulgari et al. 2011; Trivedi et al. 2012). In this study, the richness and Chao1 of phyllosphere microbiome communities decreased significantly from DM1 to DM2, while increased significantly from DM2 to DM3 (Fig. 1). Microbial communities were observed to have the lowest biodiversity at the DM2 stage, which suggested that the pathogen caused an increase in the microbial community richness when disease pressure was higher. Generally speaking, plant disease is a serious threat to indigenous microbiome biodiversity because of its inhibitory action on other microorganisms (Zhou et al. 2010a, b). The current results are in contrast with previous research (Manching et al. 2014), who found that decreased maize leaf epiphytic bacterial richness was correlated with southern leaf blight disease severity.

Significant variations were found in the microbial community structure of phyllosphere samples at different stages of angular leaf-spot of cucumber. The result of the DCA analysis indicated that the samples from different groups were clearly separated (Fig. 2). In addition, the dissimilarity test also revealed significant differences in the composition and structure of the microbiome assembled between different groups (Additional file 1: Table S1). In previous studies, It had been observed that plant pathogenic microbes shaped the microbial communities in previous researches (Zhou et al. 2010a, b; Manching et al. 2014), but it is still unclear at different disease severities. It might be due to the differences in the disease severity of plant disease, which could select the associated microorganisms to colonize leaf surface because of the symbiotic and competitive stresses among microbial species.

The composition and structure of microbial community changes with the aggravation of the disease, there were more than half of shared OTUs were observed in each disease severities group (Additional file 2: Figure S2). However, the highest number of unique OTUs of bacteria were observed in DM3 while for fungi in DM1 (Additional file 2: Figure S2). Previous studies reported that a healthy ecological environment is usually colonized more unique OTUs (Rosenzweig et al. 2012; Zhang et al. 2018). In our study, the number of unique OTUs increased from DM1 to DM2 in bacteria while decreased significantly in fungi and observed to increase significantly from DM2 to DM3 in both bacterial and fungal communities. These results indicated that plant pathogenic microbes would stimulate the growth of more antagonistic microbes in the phyllosphere. The dominating microbial phyla were the members of Proteobacteria and Ascomycota (Additional file 2: Figure S3). Proteobacteria was the most abundant bacterial population among the three disease severities (Additional file 2: Figure S3). Among fungal communities, Ascomycota were the most abundant phylum in DM1 and DM3 groups, while Basidiomycota was the most abundant phylum in DM2 group (Fig. S3). The lesion coverage rate (LCR) were negatively correlated with *Sphingomonas* and *Methylobacterium* but positively correlated with *Pseudomonas* and *Kineococcus* (p < 0.05, Table 1). It was reported that *Sphingomonas* contributes to plant health in many plants (Innerebner et al. 2011). In addition, some strains of *Pseudomonas* and *Sphingomonas* can enhance plant growth by producing plant growth hormones (Omer et al. 2004; Tsavkelova et al. 2007) and protect host plants from phytopathogens (Innerebner et al. 2011). Previous studies have also indicated that *Methylobacterium* can protect the host plants from various harmful pathogens (Ardanov et al. 2012; Madhaiyan et al. 2006). *Alternaria* is a plant pathogen causing black spots on stems and pods of *Brassica napus* (Bansal et al. 1990). Relative abundance of *Alternaria* sp. decreased with increasing lesion coverage rate (LCR), which indicated that growth of this genus may was inhibited by the angular leaf-spot causing pathogen.

The network interactions among three different groups were also analyzed in our study. Networks based on random matrix theory could accurately reflect various complex biological systems because of stronger robustness and consistency (Zhou et al. 2011). In general, the more complex network means a more stable community structure (Liang et al. 2016; Mougi and Kondoh 2012). The results of modularity values indicated that all pMENs appear to be modular (Feng et al. 2017). The modularity values for all groups were higher than those of corresponding randomized networks (Table 3). In this study, the interspecies interaction changed with the development of angular leaf-spot of cucumber. It is obvious that the bacterial network size and the interactions, especially the number of positive links (DM1: 42.16%, DM2: 61.34% and DM3: 74.4%), were significantly increased from DM1 to DM3. The results indicated that DM3 group possessed a more complex network than DM1 and DM2 group, indicating that the disease severity could affects the co-occurrence network patterns of overall bacterial communities. As previous studies described, positive and negative interactions usually mean mutualism and competition under environmental stress (Deng et al. 2016; Faust and Raes 2012). Interestingly, our results suggested that the competitive relationships between species were broken with the development of angular leaf-spot of cucumber. It was indicated that the overall bacterial community tends to mutualism from competition. The disease severity may just affects some modules. Therefore, the development of angular leaf-spot of cucumber could disrupt the stability of the microbial community network, which in turn affects ecosystem functioning.

## Additional files

**Additional file 1: Table S1.** Dissimilarity test (MRPP, ANOSIM and PERMANOVA (ADONIS)) of microorganism communities in phyllosphere from two different group. DM1, DM2 and DM3 represent the three disease severities of angular leaf-spot of cucumber, respectively. DM1: symptomatic-mild, DM2: symptomatic-moderate, DM3: symptomatic-severe. **Table S2.** The relative abundance of main microorganism genus under different disease degree. DM1, DM2 and DM3 represent the three disease severities of angular leaf-spot of cucumber, respectively. DM1: symptomatic-mild, DM2: symptomatic-moderate, DM3: symptomatic-severe. **Table S3.** Summary of module hubs and connectors in microbial communities under different disease severities. LB1, LB2 and LB3 group were the bacterial population from DM1, DM2 and DM3 disease severities. LF1, LF2 and LF3 group were the fungal population from DM1, DM2 and DM3 disease severities. Module hubs were nodes that highly connected with nodes within their modules, Zi > 2.5 and connectors were nodes that connected with several modules, Pi > 0.62.

**Additional file 2: Figure S1.** Rarefaction curve of bacterial (A) and fungal (B) communities under different disease severities. DM1: symptomatic-mild, DM2: symptomatic-moderate, DM3: symptomatic-severe. **Figure S2.** The unique and shared OTUs detected in the phyllosphere under different disease severities. DM1, DM2 and DM3 represent the three disease severities of angular leaf-spot of cucumber, respectively. DM1: symptomatic-mild, DM2: symptomatic-moderate, DM3: symptomatic-severe**. Figure S3.** Relative abundance at phylum level of bacterial and fungal communities under different disease severities. DM1, DM2 and DM3 represent the three disease severities of angular leaf-spot of cucumber, respectively. DM1: symptomatic-mild, DM2: symptomatic-moderate, DM3: symptomatic-severe. **Figure S4.** Relative abundance at class level of bacterial and fungal communities under different disease severities. DM1, DM2 and DM3 represent the three disease severities of angular leaf-spot of cucumber, respectively. DM1: symptomatic-mild, DM2: symptomatic-moderate, DM3: symptomatic-severe. **Figure S5.** Relative abundance of dominant genus of bacterial and fungal communities under different disease severities. The data were analyzed based on a one-way ANOVA followed by Duncan’s multiple range test at p < 0.05. DM1, DM2 and DM3 represent the three disease severities of angular leaf-spot of cucumber, respectively. DM1: symptomatic-mild, DM2: symptomatic-moderate, DM3: symptomatic-severe. **Figure S6.** Summary of module hubs and connectors of the bacterial (A) and fungal (B) communities under different disease severities. The OTUs were peripherals whose links mainly stayed within their respective modules. Generalists including module hubs (nodes that highly connected with nodes within their modules, Zi > 2.5) and connectors (nodes that connected with several modules, Pi > 0.62). LB1, LB2 and LB3 group were the bacterial population from DM1, DM2 and DM3 disease severities. LF1, LF2 and LF3 group were the fungal population from DM1, DM2 and DM3 disease severities.

## Data Availability

The strains were available upon request. All data obtained have been included into the manuscript and its Additional files.

## Abbreviations

LCR: lesion coverage rate
DM1: symptomatic-mild
DM2: symptomatic-moderate
DM3: symptomatic-severe
MRPP: nonparametric multi-response permutation procedures
ANOSIM: analysis of similarities
ADONIS: non-parametric permutational multivariate analysis of variance of the Adonis function
