# Supplementary material for: Variations in phyllosphere microbial community along with the development of angular leaf-spot of cucumber
Source: AMB Express. 2019 May 27;9:76. doi: 10.1186/s13568-019-0800-y (PMC6536563; doi:10.1186/s13568-019-0800-y)
Supplement: Supplementary file 1 — Additional file 1: Table S1. Dissimilarity test (MRPP, ANOSIM and PERMANOVA (ADONIS)) of microorganism communities in phyllosphere from two different group. DM1, DM2 and DM3 represent the three disease severities of angular leaf-spot of cucumber, respectively. DM1: symptomatic-mild, DM2: symptomatic-moderate, DM3: symptomatic-severe. Table S2. The relative abundance of main microorganism genus under different disease degree. DM1, DM2 and DM3 represent the three disease severities of angular leaf-spot of cucumber, respectively. DM1: symptomatic-mild, DM2: symptomatic-moderate, DM3: symptomatic-severe. Table S3. Summary of module hubs and connectors in microbial communities under different disease severities. LB1, LB2 and LB3 group were the bacterial population from DM1, DM2 and DM3 disease severities. LF1, LF2 and LF3 group were the fungal population from DM1, DM2 and DM3 disease severities. Module hubs were nodes that highly connected with nodes within their modules, Zi > 2.5 and connectors were nodes that connected with several modules, Pi > 0.62. [file 13568_2019_800_MOESM1_ESM.docx]

**Table S1** **Dissmilarity test (MRPP, ANOSIM and PERMANOVA(ADONIS)) of microorganism communities in phyllosphere from two different group.** DM1, DM2 and DM3 represent the three disease severities of angular leaf-spot of cucumber, respectively. DM1:symptomatic-mild, DM2:symptomatic-moderate, DM3: symptomatic-severe.

|  | | **MRPP** | | | | **ANOSIM** | | | | **PERMANOVA(ADONIS)** | | | |
| --- | --- | --- | --- | --- | --- | --- | --- | --- | --- | --- | --- | --- | --- |
|  |  | **Bray-Curtis** | | **Jaccard** | | **Bray-Curtis** | | **Jaccard** | | **Bray-Curtis** | | **Jaccard** | |
|  |  | **δ** | **P** | **F** | **P** | **R** | **P** | **F** | **P** | **F** | **P** | **F** | **P** |
| **Bacteria** | **DM1-DM2** | 0.5305 | 0.01 | 0.5698 | 0.03 | 0.2944 | 0.036 | 0.3425 | 0.03 | 2.2875 | 0.017 | 1.6675 | 0.035 |
|  | **DM1-DM3** | 0.5822 | 0.006 | 0.5199 | 0.004 | 0.8462 | 0.004 | 0.3287 | 0.008 | 5.0898 | 0.001 | 1.8177 | 0.002 |
|  | **DM2-DM3** | 0.5119 | 0.005 | 0.5403 | 0.004 | 0.7657 | 0.004 | 0.6796 | 0.001 | 5.9474 | 0.002 | 3.613 | 0.002 |
| **Fungi** | **DM1-DM2** | 0.466 | 0.004 | 0.6297 | 0.004 | 0.8351 | 0.005 | 0.8351 | 0.003 | 5.9994 | 0.001 | 2.4025 | 0.005 |
|  | **DM1-DM3** | 0.4616 | 0.004 | 0.6269 | 0.001 | 0.6296 | 0.003 | 0.6296 | 0.004 | 3.927 | 0.001 | 2.0982 | 0.003 |
|  | **DM2-DM3** | 0.4596 | 0.008 | 0.6246 | 0.011 | 0.237 | 0.017 | 0.237 | 0.019 | 2.2065 | 0.007 | 1.5111 | 0.005 |

**Table S2 The relative abundance of main microorganism genus under different disease degree.** DM1, DM2 and DM3 represent the three disease severities of angular leaf-spot of cucumber, respectively. DM1:symptomatic-mild, DM2:symptomatic-moderate, DM3: symptomatic-severe.

|  | **Genus** | **DM1** | **DM2** | **DM3** |
| --- | --- | --- | --- | --- |
| **Bacteria** | ***Sphingomonas*** | 29.3±6.39a | 38.86±6.99a | 11.4±3.4b |
|  | ***Microbacterium*** | 4.73±1.57b | 17.1±4.95a | 8.72±3.93ab |
|  | ***Methylobacterium*** | 22.08±5.45a | 10.86±1.54b | 2.38±0.67b |
|  | ***Curtobacterium*** | 11.46±4.54a | 3.41±1.75ab | 1.79±0.91b |
|  | ***Pseudomonas*** | 0.58±0.37b | 2.37±1.04b | 11.97±3.94a |
|  | ***Kineococcus*** | 1.52±0.55b | 1.35±0.38b | 22.91±6.63a |
|  | ***Aureimonas*** | 2.36±1.82a | 3.46±1.23a | 0.54±0.29a |
|  | ***Quadrisphaera*** | 12.68±6.15a | 1.76±0.47b | 0.2±0.04b |
|  | ***Novosphingobium*** | 0.76±0.26b | 0.31±0.12b | 3.13±0.56a |
|  | ***Hymenobacter*** | 0.41±0.17a | 0.18±0.07a | 0.17±0.09a |
|  | ***Bacillus*** | 1.06±0.38a | 0.16±0.11a | 2.25±1.4a |
|  | ***Lactobacillus*** | 0.36±0.23b | 0.01±0.01b | 3.4±0.97a |
| **Fungi** | ***Sporobolomyces*** | 33.93±4.33a | 19.64±2.28b | 9.59±2.64c |
|  | ***Davidiella*** | 9.11±1.34a | 18.25±6.37a | 8.19±1.55a |
|  | ***Phoma*** | 6.64±1.63a | 8.8±1.33a | 5.98±0.78a |
|  | ***Alternaria*** | 6.62±0.96b | 7.63±2.26b | 26.01±5.02a |
|  | ***Pseudozyma*** | 7.84±3a | 5.14±1.93ab | 0.86±0.28b |
|  | ***Aureobasidium*** | 7.4±1.89a | 3.16±0.43b | 5.84±1.05ab |
|  | ***Ascomycota_unidentified_1_1*** | 1.18±0.27a | 2.3±1.12a | 1.77±0.47a |
|  | ***Periconia*** | 0.83±0.26a | 2.27±0.78a | 1.08±0.43a |
|  | ***Exobasidiomycetes_unidentified_1*** | 1.77±0.99a | 0.88±0.22a | 1.4±0.7a |
|  | ***Pleosporales_unidentified_1*** | 0.34±0.07a | 0.84±0.11a | 2.24±1.06a |
|  | ***Tremellomycetes_unidentified_1*** | 2.54±1.46a | 0.34±0.1a | 1.51±0.43a |
|  | ***Chaetothyriales_unidentified_1*** | 0.08±0.02a | 0.2±0.04a | 2.78±1.53a |
|  | ***Arthrinium*** | 0.2±0.12b | 0.19±0.04b | 2.01±0.54a |

**Table S3 Summary of module hubs and connectors in microbial communities under different disease severities.** LB1, LB2 and LB3 group were the bacterial population from DM1, DM2 and DM3 disease severities. LF1, LF2 and LF3 group were the fungal population from DM1, DM2 and DM3 disease severities. Module hubs were nodes that highly connected with nodes within their modules, Zi > 2.5 and connectors were nodes that connected with several modules, Pi > 0.62.

| **Hubs** | **Group** | **Name** | **Phylum** | **Genus** | **Relative abundance** |
| --- | --- | --- | --- | --- | --- |
| **Module hubs(Bacteria)** | **LB1** | OTU_44 | *Bacteroidetes* | *Sphingobacterium* | 1.14E-03 |
|  | **LB2** | OTU_271 | *Bacteroidetes* | *Spirosoma* | 4.89E-05 |
|  |  | OTU_282 | *Proteobacteria* | *Pantoea* | 3.03E-03 |
|  |  | OTU_86 | *Firmicutes* | *Bacillus* | 7.99E-04 |
| **Connectors(Bacteria)** | **LB1** | OTU_20 | *Firmicutes* | *Melissococcus* | 7.03E-03 |
|  |  | OTU_45 | *Proteobacteria* | *Aureimonas* | 2.82E-03 |
|  |  | OTU_73 | *Actinobacteria* | *Nocardioides* | 2.12E-04 |
|  |  | OTU_252 | *Firmicutes* | *Bacillus* | 1.14E-04 |
|  | **LB2** | OTU_5 | *Actinobacteria* | *Curtobacterium* | 3.41E-02 |
|  |  | OTU_15 | *Actinobacteria* | *Microbacterium* | 1.04E-02 |
|  |  | OTU_101 | *Proteobacteria* | *Devosia* | 4.73E-04 |
|  | **LB3** | OTU_125 | *Proteobacteria* | *Massilia* | 3.43E-04 |
|  |  | OTU_150 | *Actinobacteria* | *Rhodococcus* | 1.31E-04 |
|  |  | OTU_226 | *Proteobacteria* | *Brevundimonas* | 1.14E-04 |
|  |  | OTU_69 | *Firmicutes* | *Clostridium sensu stricto 1* | 9.95E-04 |
| **Module hubs(Fungi)** | **LF1** | OTU_834 | *Basidiomycota* | *Exobasidium* | 3.75E-05 |
|  | **LF2** | OTU_7 | *Basidiomycota* | *Bulleromyces* | 8.78E-03 |
|  |  | OTU_36 | *Ascomycota* | *Leptospora* | 6.87E-04 |
|  |  | OTU_105 | *Basidiomycota* | *Cryptococcus_1* | 2.24E-04 |
|  | **LF3** | OTU_33 | *Ascomycota* | *Strelitziana* | 1.39E-03 |
|  |  | OTU_376 | *Ascomycota* | *Pleosporales_unidentified_1* | 8.74E-05 |
|  |  | OTU_380 | *Ascomycota* | *Sordariomycetes_unidentified_1* | 7.50E-05 |
| **Connectors(Fungi)** | **LF1** | OTU_188 | *Ascomycota* | *Devriesia* | 4.37E-04 |
|  |  | OTU_450 | *Ascomycota* | *Monochaetia* | 1.12E-04 |
|  | **LF2** | OTU_61 | *Ascomycota* | *Ascomycota_unidentified_1_1* | 3.50E-04 |
|  | **LF3** | OTU_23 | *Ascomycota* | *Davidiella* | 2.89E-02 |
|  |  | OTU_14 | *Ascomycota* | *Ascomycota_unidentified_1_1* | 1.61E-02 |
|  |  | OTU_35 | *Ascomycota* | *Didymella* | 1.51E-03 |
|  |  | OTU_193 | *Ascomycota* | *Physciella* | 2.12E-04 |
|  |  | OTU_184 | *Ascomycota* | *Unclassified* | 1.62E-04 |
