# Supplementary material for: Variations in phyllosphere microbial community along with the development of angular leaf-spot of cucumber
Source: AMB Express. 2019 May 27;9:76. doi: 10.1186/s13568-019-0800-y (PMC6536563; doi:10.1186/s13568-019-0800-y)
Supplement: Supplementary file 2 — Additional file 2: Figure S1. Rarefaction curve of bacterial (A) and fungal (B) communities under different disease severities. DM1: symptomatic-mild, DM2: symptomatic-moderate, DM3: symptomatic-severe. Figure S2. The unique and shared OTUs detected in the phyllosphere under different disease severities. DM1, DM2 and DM3 represent the three disease severities of angular leaf-spot of cucumber, respectively. DM1: symptomatic-mild, DM2: symptomatic-moderate, DM3: symptomatic-severe. Figure S3. Relative abundance at phylum level of bacterial and fungal communities under different disease severities. DM1, DM2 and DM3 represent the three disease severities of angular leaf-spot of cucumber, respectively. DM1: symptomatic-mild, DM2: symptomatic-moderate, DM3: symptomatic-severe. Figure S4. Relative abundance at class level of bacterial and fungal communities under different disease severities. DM1, DM2 and DM3 represent the three disease severities of angular leaf-spot of cucumber, respectively. DM1: symptomatic-mild, DM2: symptomatic-moderate, DM3: symptomatic-severe. Figure S5. Relative abundance of dominant genus of bacterial and fungal communities under different disease severities. The data were analyzed based on a one-way ANOVA followed by Duncan’s multiple range test at p < 0.05. DM1, DM2 and DM3 represent the three disease severities of angular leaf-spot of cucumber, respectively. DM1: symptomatic-mild, DM2: symptomatic-moderate, DM3: symptomatic-severe. Figure S6. Summary of module hubs and connectors of the bacterial (A) and fungal (B) communities under different disease severities. The OTUs were peripherals whose links mainly stayed within their respective modules. Generalists including module hubs (nodes that highly connected with nodes within their modules, Zi > 2.5) and connectors (nodes that connected with several modules, Pi > 0.62). LB1, LB2 and LB3 group were the bacterial population from DM1, DM2 and DM3 disease severities. LF1, LF2 and LF3 group were the [file 13568_2019_800_MOESM2_ESM.docx]

**
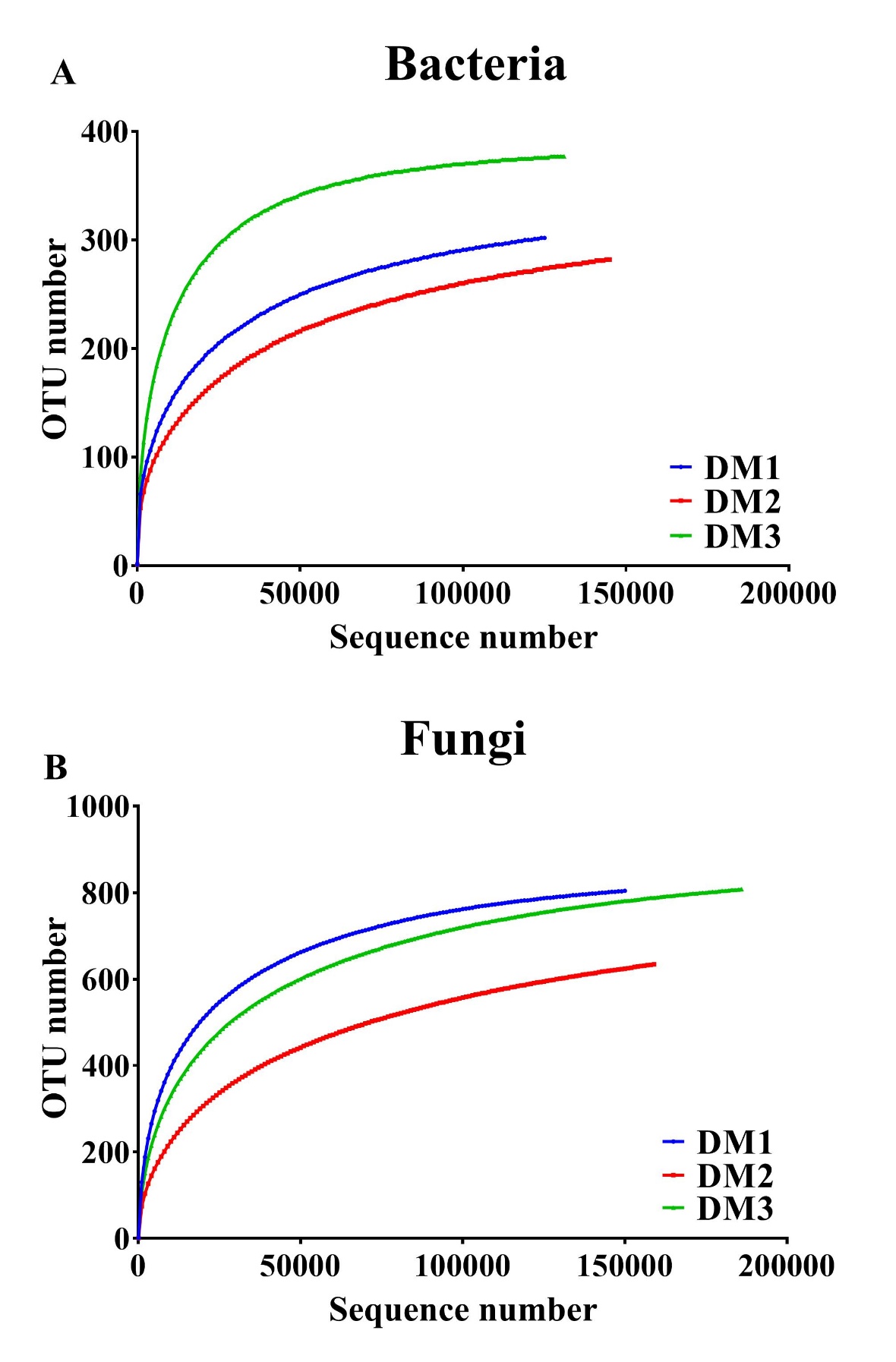
**

**Figure S1 Rarefaction curve of bacterial (A) and fungal (B) conmmunities under different disease severities.** DM1:symptomatic-mild, DM2:symptomatic-moderate, DM3: symptomatic-severe**.**

**
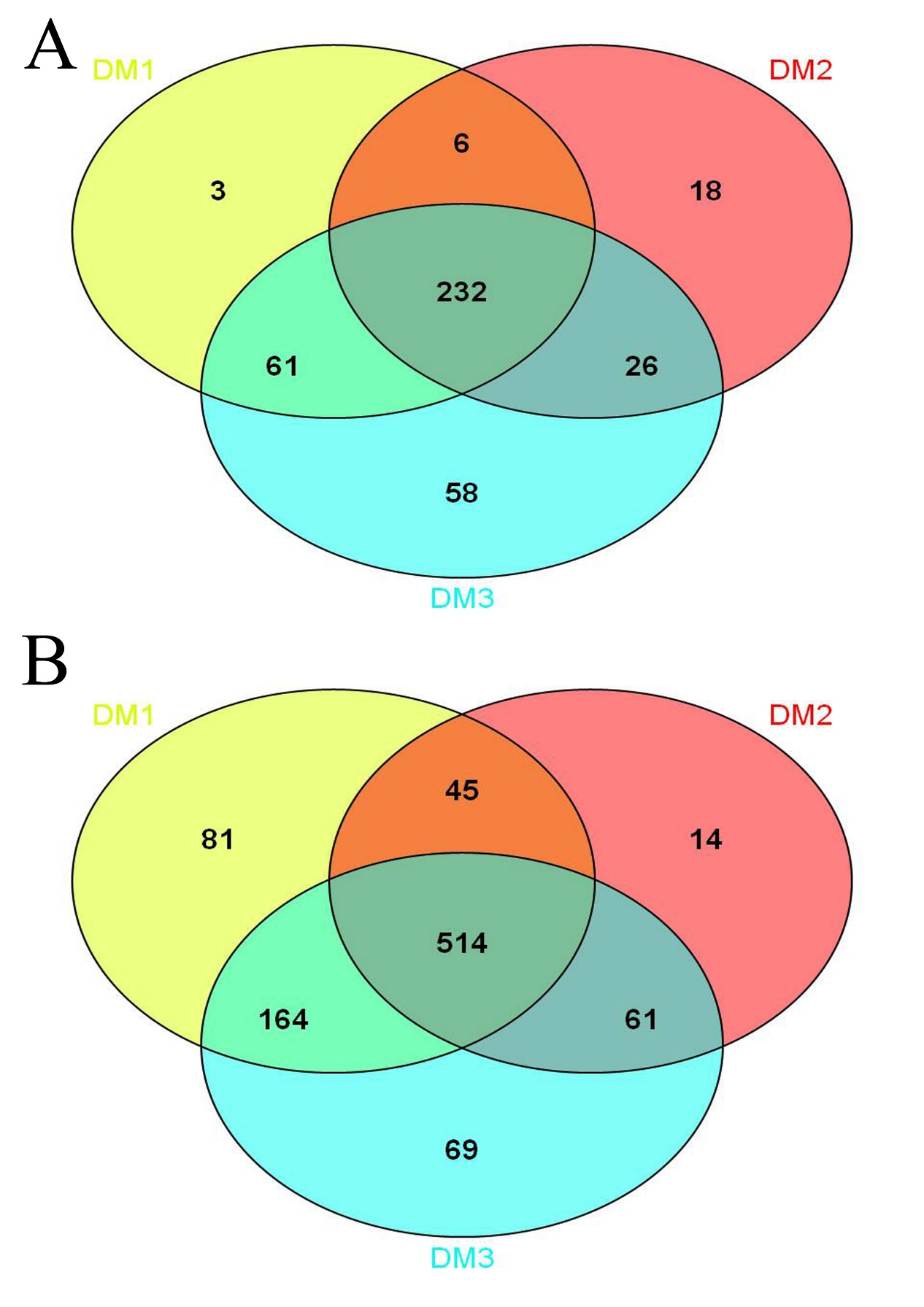
**

**Figure S2 The unique and shared OTUs detected in the phyllosphere under different disease severities.** DM1, DM2 and DM3 represent the three disease severities of angular leaf-spot of cucumber, respectively. DM1:symptomatic-mild, DM2:symptomatic-moderate, DM3: symptomatic-severe**.**

**
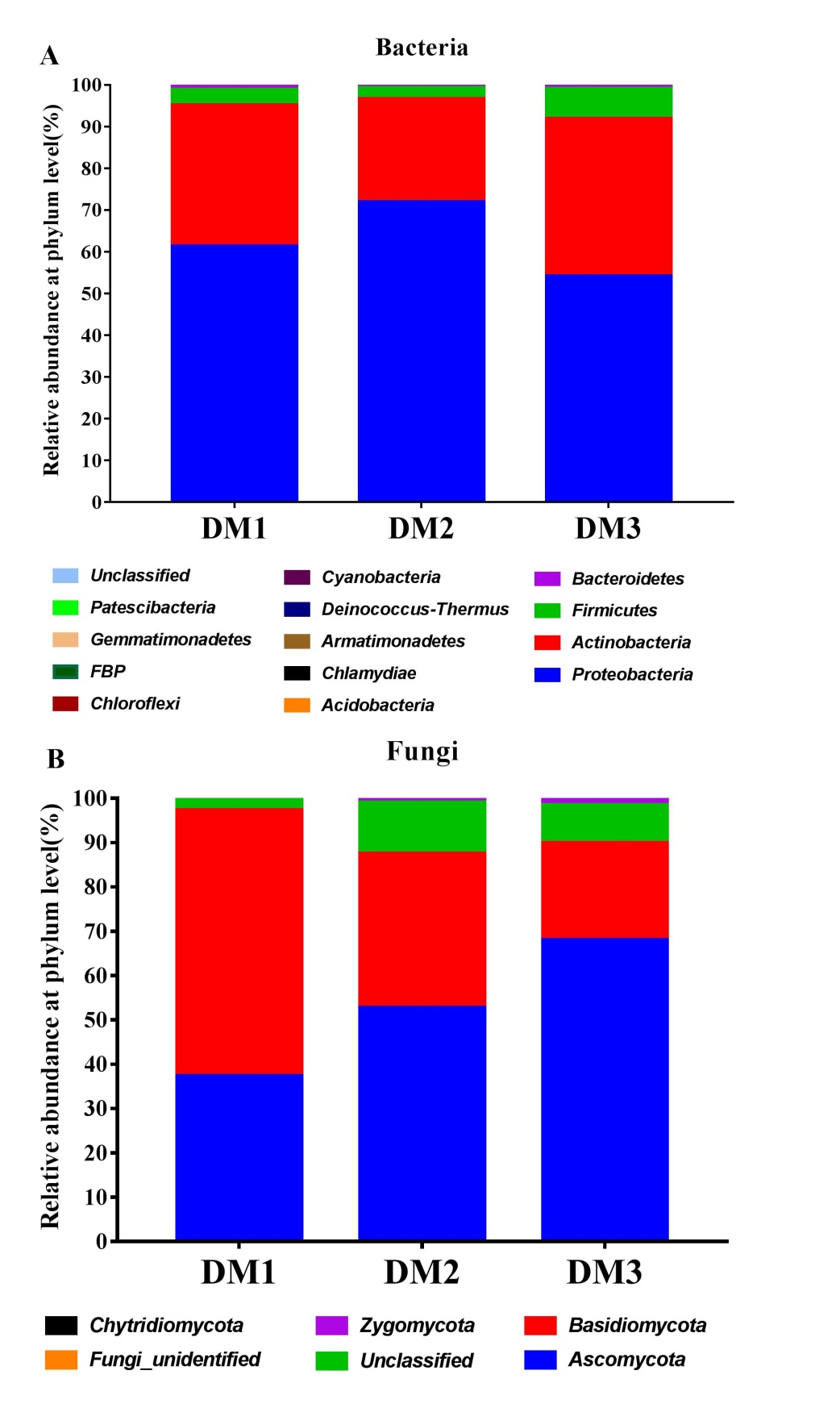
**

**Figure S3 Relative abundance at phylum level of bacterial and fungal communities under different disease severities.** DM1, DM2 and DM3 represent the three disease severities of angular leaf-spot of cucumber, respectively. DM1:symptomatic-mild, DM2:symptomatic-moderate, DM3: symptomatic-severe**.**

**
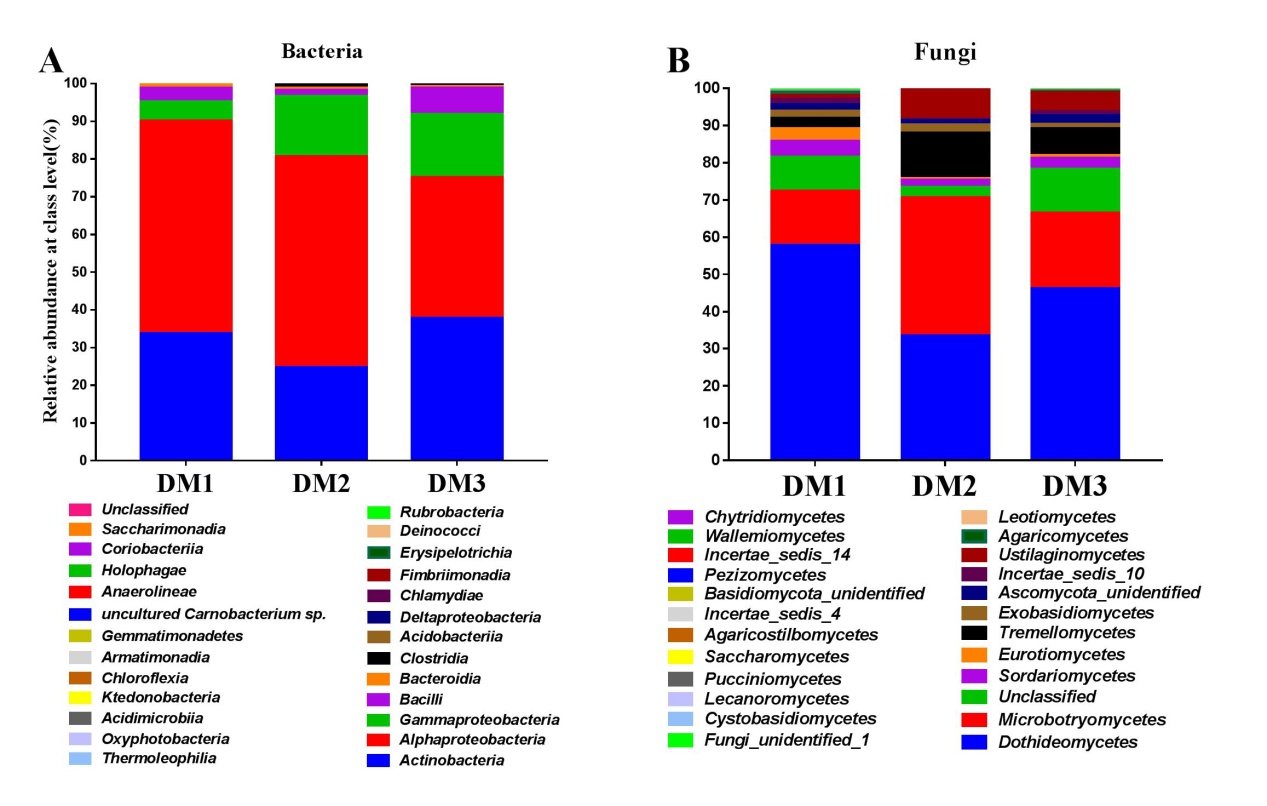
**

**Figure S4 Relative abundance at class level of bacterial and fungal communities under different disease severities.** DM1, DM2 and DM3 represent the three disease severities of angular leaf-spot of cucumber, respectively. DM1:symptomatic-mild, DM2:symptomatic-moderate, DM3: symptomatic-severe**.**

**
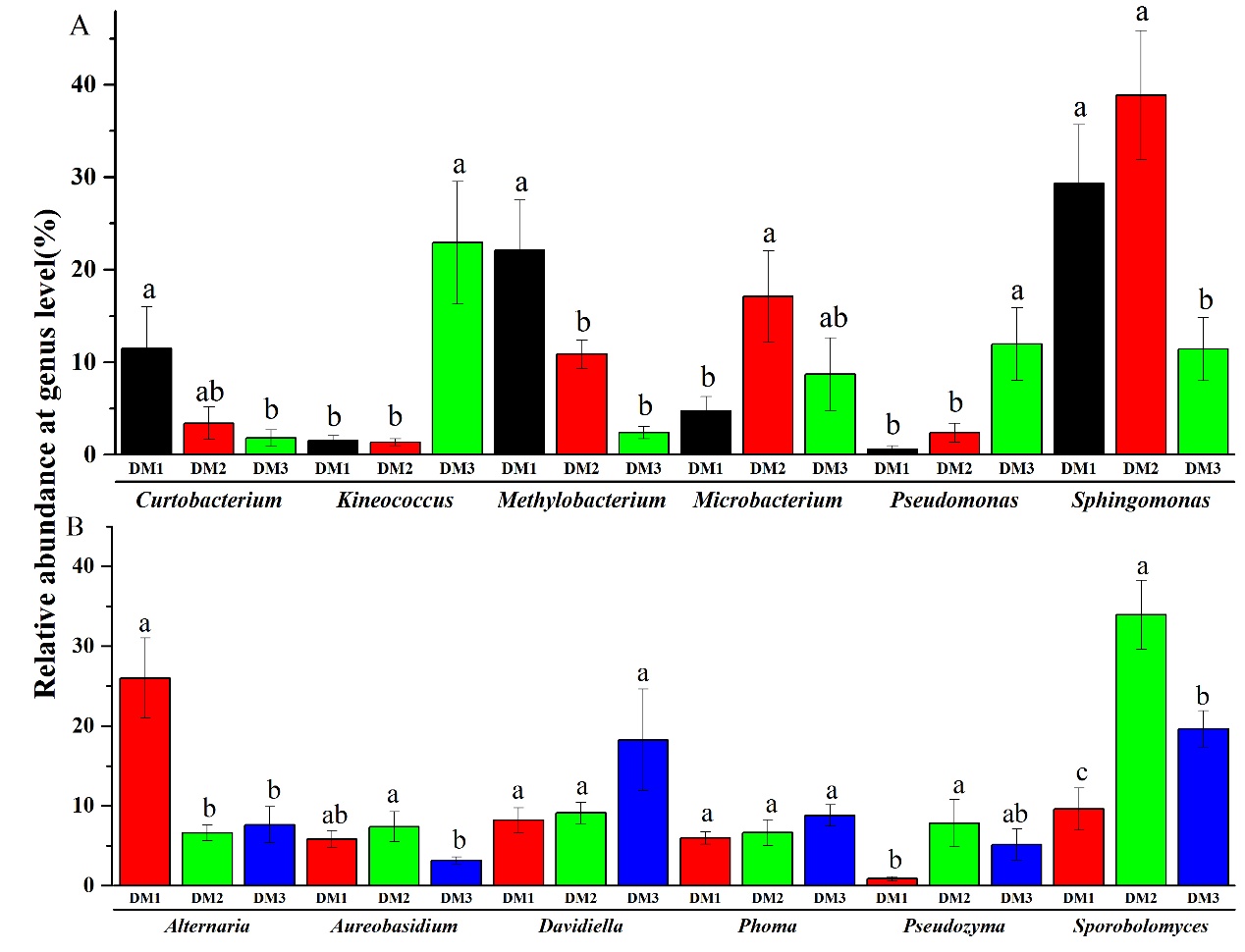
**

**Figure S5 Relative abundance of dominant genus of bacterial and fungal communities under different disease severities.** The data were analyzed based on a one-way ANOVA followed by Duncan's multiple range test at p < 0.05. DM1, DM2 and DM3 represent the three disease severities of angular leaf-spot of cucumber, respectively. DM1:symptomatic-mild, DM2:symptomatic-moderate, DM3: symptomatic-severe**.**

**
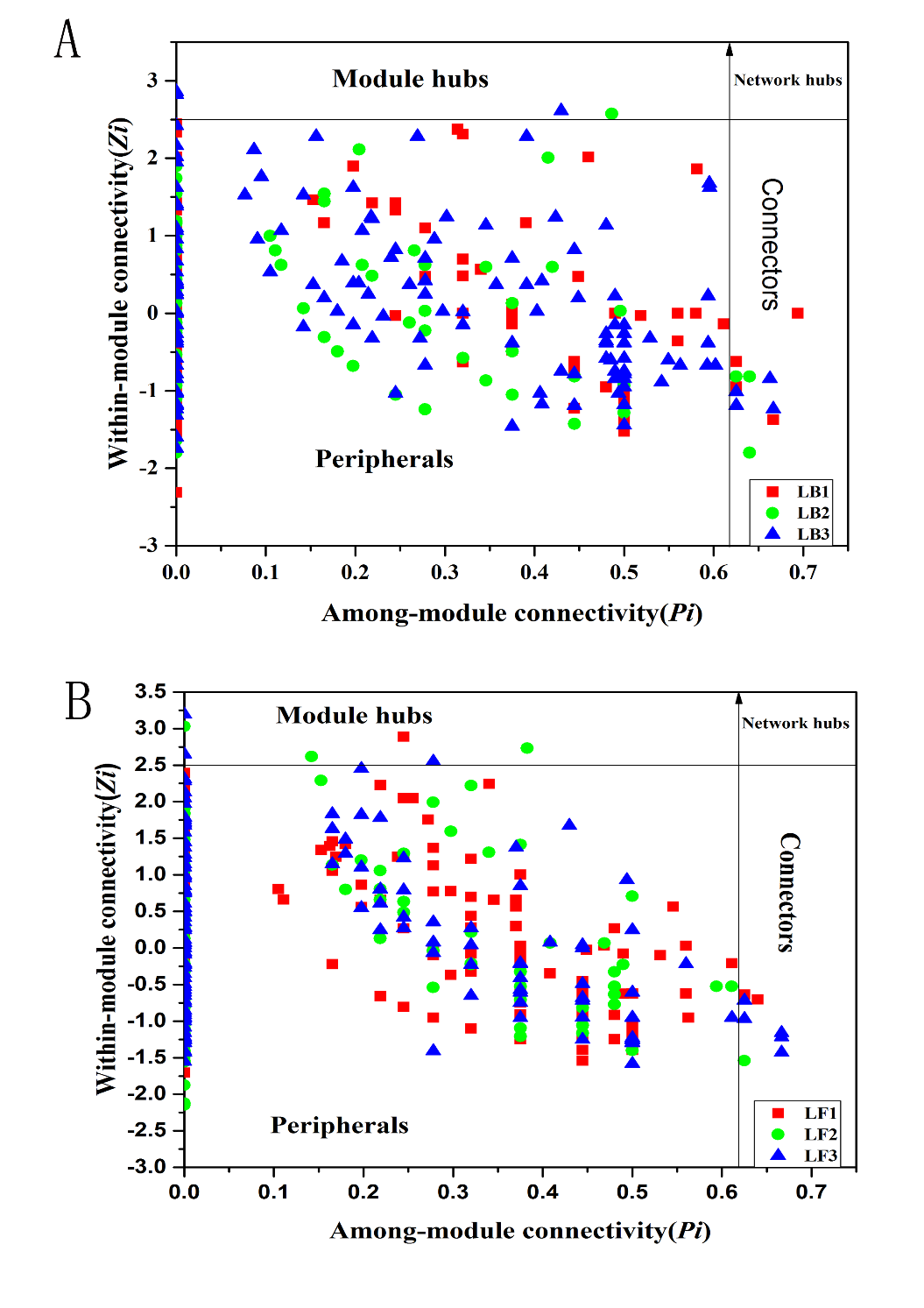
**

**Figure S6 Summary of mudule hubs and connectors of the bacterial (A) and fungal (B) communities under different disease severities.** The OTUs were peripherals whose links mainly stayed within their respective modules. Generalists including module hubs (nodes that highly connected with nodes within their modules, Zi > 2.5) and connectors (nodes that connected with several modules, Pi > 0.62). LB1, LB2 and LB3 group were the bacterial population from DM1, DM2 and DM3 disease severities. LF1, LF2 and LF3 group were the fungal population from DM1, DM2 and DM3 disease severities.
